# MiR-141 Suppresses the Migration and Invasion of HCC Cells by Targeting Tiam1

**DOI:** 10.1371/journal.pone.0088393

**Published:** 2014-02-13

**Authors:** Ying Liu, Yi Ding, Jing Huang, Shuang Wang, Wen Ni, Jian Guan, Qisheng Li, Yuqin Zhang, Yanqing Ding, Bin Chen, Longhua Chen

**Affiliations:** 1 Department of Radiation Oncology, Nanfang Hospital, Southern Medical University, Guangzhou, Guangdong Province, PR China; 2 General Hospital of Guangzhou Military Command of PLA, Southern Medical University, Guangzhou, Guangdong Province, People’s Republic of China; 3 Department of Pathology, School of Basic Medical Sciences, Southern Medical University, Guangzhou, Guangdong Province, People’s Republic of China; 4 Department of Cancer Center of Affiliated Hospital, Guangdong Medical College, Zhanjiang, China; University of North Carolina School of Medicine, United States of America

## Abstract

**Background:**

We have demonstrated that T lymphoma invasion and metastasis 1 (Tiam1) gene is associated with the poor prognosis of patients with hepatocellular carcinoma (HCC), and we used a computational approach to identify miR-141 as a Tiam1-targeting microRNA (miRNA). Here, we explored the function of miR-141 and the relationship between miR-141 and Tiam1 gene in HCC.

**Methods:**

The miR-141 expression in HCC tissues and cell lines was detected and its roles in regulation of HCC cell proliferation, migration and invasion and target gene expression was investigated. Tiam1 was identified as a novel target of miR-141. Ethics statement: our study was approved by the Nanfang Hospital Medical Ethics Committee Ethics statement. Written informed consent was obtained before collection.

**Results:**

Based on in situ hybridization (ISH) analysis, miR-141 was down-regulated in the same HCC samples. Kaplan-Meier analysis demonstrated that patients with low miR-141 expression had poorer overall survival rate than that of the patients with high miR-141 expression. Furthermore, multivariate Cox regression analysis indicated that miR-141 could serve as an independent prognostic factor in HCC. MiR-141 significantly inhibited in vitro cell proliferation, migration and invasion as proved by gain- and loss- of function studies, while the mRNA and protein levels of Tiam1 were reduced in cells over-expressing miR-141. Moreover, Tiam1 treatment antagonized this effect, while knockdown of Tiam1 by Tiam1 short hairpin RNA (shTiam1) induced inhibitory effects.

**Conclusions:**

These findings indicated that miR-141 functions as a tumor suppressor and inhibits the migration and invasion of HCC cells by targeting Tiam1, which may provide novel prognostic and treatment strategies for HCC patients.

## Introduction

Hepatocellular carcinoma (HCC) is the sixth most common cancer and the third cause of cancer-related death, resulting in approximately 600,000 to 1,000,000 deaths annually in the world [1], [2]. Currently, surgical resection and transplantation are the effective treatment approaches for hepatocellular carcinoma [3]. However, the recurrence rate within 2 years in patients who have undergone of tumor resection remains more than 50% [4], [5]. Uncontrolled tumor metastasis, frequent intrahepatic spread and extrahepatic metastasis are the primary causes for the poor prognosis of HCC [6]. Therefore, improved understanding of the molecular mechanisms of HCC invasion and metastasis is essential for the development of new therapeutic strategies.

MiRNAs are a class of small, endogenous and noncoding RNAs, regulating gene expression by binding to sequences in a 3′ untranslated region (3′UTR) of target mRNA, resulting in translational repression and/or degradation of the mRNA [7]. Growing evidence indicates that abnormal expression/function of miRNAs contributes to tumorigenesis and carcinoma progression of various human cancers [8].

Tiam1, encodes a 177-kDa protein that is a member of the Dbl family of guanine nucleotide exchange factor (GNEF) that regulate small G proteins of the Rho family [9], [10]. The relationship between Tiam1 and metastasis was first identified in T-lymphoma cells in 1994 [11]. Accordingly, Tiam1 has been shown to act as a metastasis-related gene in a variety of cancers, including breast cancer [12], [13], colorectal cancer (CRC) [14], [15], prostate cancer [16], lung cancer [17], Ras-induced skin tumors [18] and renal cell carcinoma [19]. In our previous study, we found that Tiam1 not only correlated with a poor prognosis in patients with HCC but also contributed to HCC invasion and metastasis [20], [21]. However, the underlying molecular mechanisms of its activities in HCC have yet to be fully elucidated. Thus, modulators of Tiam1 gene expression, such as miRNAs, may be predicted to have a profound effect on tumor progress. Recent studies have identified that Tiam1 is a functional target of miR-10b, miR-21 and miR-31 in different cancers [22], [23], revealing the miRNA regulatory networks on Tiam1 expression.

In this study, we first used publicly available databases to identify miR-141 as a Tiam1-targeting miRNA, and we found that the expression of miR-141 and Tiam1 was inversely correlated in HCC cells. Therefore, we evaluated the expression profile of miR-141 in different human HCC cell lines and confirmed the regulatory effect of miR-141 on Tiam1 and its function in HCC which may provide a novel candidate target for therapeutic strategies in HCC.

## Materials and Methods

### Patients and Tissue Samples

We used the same 212 HCC samples from those enrolled in our previous study who had undergone routine surgery at Nangfang Hospital and Zhujiang Hospital, Guangzhou City, Guangdong Province, China between 1999 and 2002, They were not pretreated with radiotherapy or chemotherapy prior to surgery [21]. Samples intended for later in situ hybridization (ISH) analyses followed routine fixation and paraffin embedding in an RNase-free environment. Another primary HCC tissue samples and matched adjacent non-tumor samples were obtained randomly from 30 patients undergoing hepatectomy at Nangfang Hospital, Guangzhou, Guangdong, China. Written informed consent was obtained before collection. Samples were immediately snapped frozen and stored in liquid nitrogen for RNA analysis. The histological types were assigned according to the criteria of the WHO classification system.

### Locked Nucleic Acid in situ Hybridization (LNA ISH)

LNA ISH on paraffin tissue sections with a double DIG-labeled Locked Nucleic Acid (LNA) probe specific for human miR-141 was performed according to the manufacturer’s instructions (Exiqon, Woburn, MA). In brief, Sections (4 cm) of archived paraffin-embedded specimens were deparaffinized in xylenes and then rehydrated through an ethanol dilution series (from 99.9% to 70%). Sections were treated with proteinase K at 37°C for 10 minutes and then dehydrated through an ethanol dilution series (from70% to 99.9%). Slides were incubated in a DIG-labeled probe diluted to 250 nM in an hybridization buffer at 50°C for 2 hours. Stringent washes were performed with 5×SSC, 1×SSC and 0.2×SSC buffers at 50°C over 33 min, DIG blocking reagent (Roche) in maleic acid buffer containing 2% sheep serum at 30°C for 15 min, alkaline phosphatase-conjugated anti-digoxigenin (diluted 1∶500 in blocking reagent, Roche) at room temperature for 60 min, enzymatic development using 4-nitro-blue tetrazolium (NBT) and 5-brom-4-chloro-30-Indolyl-phosphate (BCIP) substrate (Roche) forming dark-blue NBT-formazan precipitate at 30°C for 120 min, followed by nuclear fast counterstain for 5 min. The slides were then dismantled in water, dehydrated in alcohol solutions and mounted with eukitt mounting medium (VWR, Herlev, Denmark). Scrambled probe was detected as a control. Signals were visually quantified using a quick score system from 0 to 5, combining intensity of signal and percentage of positive cells (signal: 0 =  no signal, 1 =  weak signal, 2 =  intermediate signal, 3 =  strong signal; percentage: 0 = 0%, 1 = <30%, 2 = >30%) [24]. Tissue sections were blindly examined by a second individual and this yielded a good agreement with the initial quantifications.

### Cell Lines and Culture Conditions

The two human HCC cell lines, MHCC97L and HCCLM3 were obtained from the Liver Cancer Institute and the Zhongshan Hospital of Fudan University, Shanghai, China. These two clonal cell strains were derived from the same parent cell MHCC97 to ensure a similar genetic background and yet yielded dramatic differences in spontaneous metastatic behavior. Compared with MHCC97L, the HCCLM3 cell line was established from nude mouse lung metastasis, which was characterized by high pulmonary metastasis via both subcutaneous and orthotopic inoculati. They were maintained in low glucose DMEM (Gibco) supplemented with 10% fetal bovine serum (Hyclone). All the media were supplemented with 100 U/ml penicillin and 100 µg/ml streptomycin (Invitrogen) and incubated at 37°C in a humidified chamber containing 5% CO2. An immortalized normal hepatocyte cell line, LO2, and 293FT cells which purchased from the cell bank of Chinese Academy of Sciences were also maintained in DMEM (Gibco) supplemented with 10% FBS.

### RNA Extraction and Reverse Transcription PCR

Total RNA was extracted using TRIzol Reagent (Takara, Dalian, China). For miRNA analysis, cDNA was synthesized using the All-in-One™ miRNA First-Strand cDNA synthesis kit (GeneCopoeia). Quantitative PCR was undertaken in the following thermo-cycler conditions: 95°C for 10 min, and 40 cycles of 95°C for 10 s and 60°C for 20 s and 72°C for 34 s. The primer of mature miR-141 was synthesized by GeneCopoeia (ID hsmq-0596); U6 was used as an internal control. For Tiam1 analysis, cDNA was synthesized according to the manufacturer’s protocol (reverse transcriptase cDNA synthesis kit, Takara, Dalian, China). RT-PCR analysis was performed using an SYBR PrimeScript RT-PCR Kit (Takara) according to the manufacturer’s protocol. The forward primer 5′-AAGACGTACTCAGGCCA- TGTCC-3′ and the reverse primer 5′-GACCCAAATGTCGCAGTCAG-3′ were used to amplify a 252-bp PCR product for human Tiam1 (GeneBank, NM_003253). Experiments were repeated at least three times to ensure the reproducibility of the results. Human β-actin gene was amplified as an endogenous control. Comparative quantification was determined using the 2^−ΔΔCt^ method [25].

### Establishment of miR-141 Over-expressed Cells

HCCLM3 cells were plated to reach 50% confluence after 24 hours; miR-141 over-expressed lentivirus and negative control lentivirus (Sunbio medical biotechnology Co., Ltd, Shanghai, China) containing polybrene (8 g/ml) were added to cells. After 6 hours of incubation, the medium was exchanged for fresh 10% FBS according to the cells’ state. Flow cytometry assays were applied to sort the cells expressing GFP and their corresponding negative control. These two groups of cells were identified by real-time PCR. The cell expressing the highest level of miR-141 was termed M3/miR-141+, whilst the negative one was termed M3/mock.

### Oligonucleotide Transfection

The MHCC97L cell was transfected with 2-O-methyl anti-miR-141 inhibitor or negative control (100 pmol, GenePharma) using Lipofectamine2000 (Invitrogen). The expression of miR-141 was validated by real-time PCR. The cell cells were termed 97L/inhibitor, 97L/control, respectively; and the M3/miR-141+ cell was transfected with p-EZ-M02-Tiam1 plasmid or p-EZ-M02 vector (2 µg, FulenGen) or Tiam1 short hairpin RNA (shTiam1) or negative control (150 pmol, GenePharma) using Lipofecta- mine 2000 reagent (Invitrogen).

### Western Blot

Total protein was extracted using RIPA lysis buffer and total protein samples (30 µg) were separated using 8% polyacrylamide SDS gels and transferred to polyvinylidene fluoride membranes (Millipore). The membranes were incubated with rabbit polyclonal anti-Tiam1 antibody (1∶200; Santa Cruz) followed by horseradish peroxidase -conjugated goat anti-rabbit IgG (1∶5000; Ebiogenes) and the bands were detected using enhanced chemiluminescence. A mouse anti-beta actin moloclonal antibody (1∶500; ZSGB-BIO) was used as a loading control.

### CCK-8 Assay

Cells were plated in 96-well plates at 1×10^3^ cells per well. After incubation for one day, 10 µl of the CCK-8 solution was added to each well and incubation continued for 2 h. The absorbance was measured at 450 nm using Enspire™ multilable reader (Tueku, Filand). All experiments were performed in triplicate.

### Wound-healing Assay

Cells were plated in a 6-well plate. When cell confluence reached approximately 90%, wounds were created in mono-layers of cells using a 10 µl pipette tip. Cells were washed to remove cellular debris and incubated at 37°C. Images were taken at different points of time following wounding. Duplicate wells for each condition were examined for each experiment and each experiment was repeated three times. The percentage of the wound healing was calculated as (the width of wound at 0 h - the width of wound at 72 h)/the width of wound at 0 h.

### Transwell Invasion Assay

Target HCC cells (10^5^/200 µL) were plated to the upper chamber (BD Bioscience) in serum-free medium with the lower chamber filled with 10% fetal bovine serum gradient and incubated for 24–48 h at 37°C in a 5%CO_2_ humidified chamber. After being washed twice with PBS, cells that remained on the top of the filter were removed using wet cotton swabs and fixed in methanol for 10 mins and stained with hematoxylin for 30 min. Whole filters were manually counted under the inverted microscope in five random fields (×100), and the average value was calculated.

### Dual-Luciferase Reporter Assay

293FT cells were cultured in 24-well plates and each was transfected with 0.5 ug Plasmid containing psiCHECK-2/Tiam1 or psiCHECK-2/Tiam1-mut together with Renilla and Firefly luciferase and 1 µl lipofectamine2000 50 nM and mature-miR-141 or 100 nM miR-141-inhibitor. Forty-eight hours after transfection, cells were harvested and assayed with a Dual-Luciferase Reporter assay kit (Promega) according to the manufacturer’s instructions. Each transfection was repeated three times.

### Statistical Analysis

SPSS 13.0 software was used for statistical analysis. All results were presented as the mean ± SEM. RT-PCR, clone formation, CCK-8 analysis and in vitro invasion assay were examined using one-way ANOVA. Spearman’s correlation was used to analyze the correlation between miR-141 and Tiam1 expression. The correlations of miR-141 expression to various clinicopathological parameters were evaluated with χ^2^ test. The Kaplan–Meier method and log-rank test were used to estimate survival; hazard ratios (HR) were calculated using unadjusted univariate Cox regression analysis. Multivariate Cox regression analysis was used to test for independent prognostic factors. A p value less than 0.05 was considered statistically significant.

## Results

### Expression of miR-141 in HCC by LNA ISH and Association with Patients’ Survival

**Figure 1:**
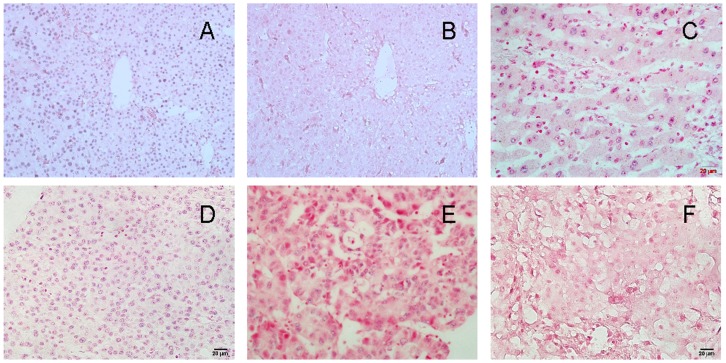
In situ hybridization (ISH) analysis of miR-141 expression in HCC tissues and surrounding noncancerous tissues. (A) Positive signals (U6) stain blue in nuclei in HCC (×400). (B) Negative (scramble-miR) in HCC (×400). (C) Moderate miR-141 staining in normal liver tissues (×400). (D) Strong staining in HCC (×400). (E) Weak staining in HCC (×400). (F) Negative staining in HCC tissue (×400). ISH positive signals (miR-141) stain blue in cellular nucleus and cytoplasm.

To examine the clinical relevance of miR-141 in HCC, its expression was analyzed by LNA ISH. As a whole, the miR-141 expression was weak in HCC tissues since the positive signals were detected until the miR-141 probe was 5 times higher than the reference concentration used. Of 212 HCC tissue samples, 90 (42.4%) had a high expression of miR-141 (score 3 to 5) and 122 (57.6%) a low expression (score 0 to 2). As shown in Figure 1, the miR-141 was detected at variable levels and localized in the cellular nucleus and cytoplasm (Figure 1). Patients with metastasis had a higher likelihood of low miR-141 expression (39 of 56, 69.6%) compared with those without metastasis (83 of 156, 53.2%). No statistically significant relationships were found between miR-141 expression and any of the clinicopathological parameters except for metastasis (p = 0.027) (Table 1).

**Table 1:** Relationship between miR-141 expression and clinicopathological features of HCC patients.

		MiR-141expression			
Features	n	High	low	*P* value	?^2^
All cases	212	90	122		
Age				0.866	0.028
**<50**	114	49	65		
**≥50**	98	41	57		
Gender				0.637	0.222
**Male**	152	63	89		
**Female**	60	27	33		
Tumor size (cm)				0.083	2.996
**<5**	80	40	40		
**≥5**	132	50	82		
Histological				0.120	4.244
differentiation					
**Well**	29	14	15		
**Moderate**	100	48	52		
**Poor**	83	28	55		
Liver cirrhosis				0.104	2.636
**Yes**	97	47	50		
**No**	115	43	72		
Metastasis				0.027^1^	4.906
**Yes**	56	17	39		
**No**	156	73	83		
Recurrence				0.479	0.501
**Yes**	79	36	43		
**No**	133	54	79		
HBsAg status				0.874	0.025
**Positive**	166	70	96		
**Negative**	46	20	26		
Serum AFP (ng/ml)				0.246	1.344
**<25**	64	31	33		
**≥25**	148	59	89		

1 Statistically significant (*P*<0.05).

**Figure 2:**
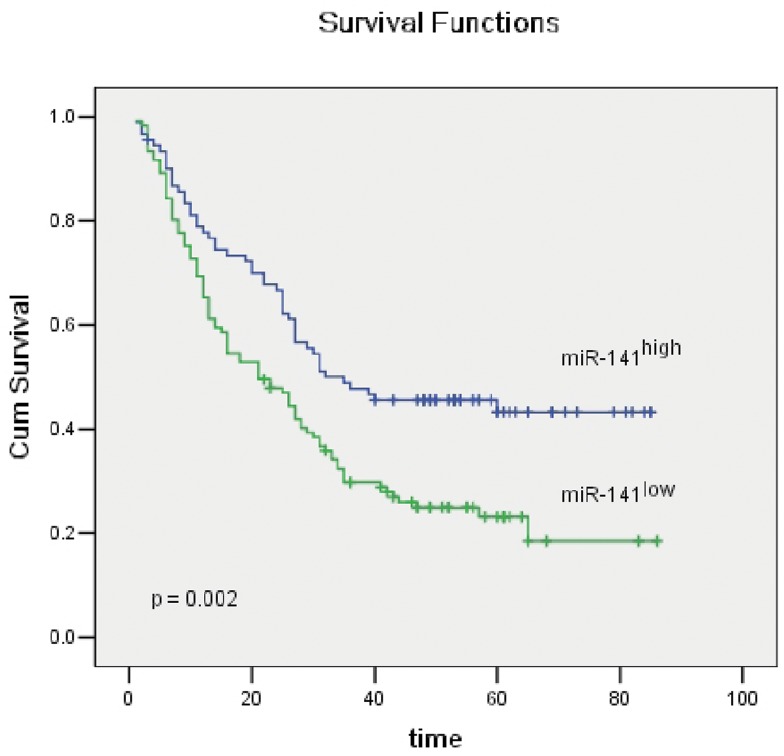
Kaplan-Meier survival analysis of primary HCC patients (n = 212) after surgical resection with high miR-141 expression (n = 90) and low miR-141 expression (n = 122). The survival rate for patients in the low-miR-141 group was significantly lower than that for patients in the high-miR-141group (log rank, *P* = 0.002).

The prognostic effect of miR-141 on HCC patients’ overall survival between patients with high and low miR-141 expression levels was compared using the Kaplan-Meier curve assessment. It was observed that a significant separation between low ISH expressions versus high ISH expressions of miR-141 in the 212 HCC patients occurred (Figure 2, p = 0.002, log-rank test), which indicated that low miR-141 expression was a significant prognostic factor for poor overall survival in HCC patients.

### Univariate and Multivariate Analyses of Prognostic Variables in HCC Patients

To identify the variables of potential prognostic significance in all the patients with HCC, we performed univariate analysis to explore the relationship of each variable with the survival time. The ratio hazard and p value for each variable were used to assess the difference in predicting the prognosis. Then, multivariate Cox proportional hazards model analysis was performed to identify the relative importance of each variable. The univariate analysis showed that the significant prognostic factors were miR-141 expression, tumor size, tumor grade, recurrence, metastasis and serum AFP. Multivariate analysis results showed that miR-141 expression, tumor size, tumor grade, metastasis and serum AFP might play a role in predicting the overall survival in HCC patients (Table 2).

**Table 2:** Univariate and multivariate analysis of individual parameters for correlations with overall survival rate cox proportional hazards model.

variables	univariate		*P* value	multivariate		*P* value
	HR CI (95%)			HR CI (95%)		
MiR-141	0.671	0.468–0.963	0.030^1^	1.724	1.219–2.439	0.002^1^
Age	0.910	0.639–1.295	0.600			
Gender	0.874	0.589–1.298	0.506			
Tumor size	2.681	1.777–4.043	0.0002^1^	0.324	0.220–0.467	0.0001^1^
Tumor grade (differentiation)			0.047^1^	1.690	1.310–2.181	0.0006^1^
Liver cirrhosis	1.586	1.088–2.313	0.017^1^	1.275	0.916–1.774	0.150
HBsAg status	1.020	0.647–1.609	0.933			
metastasis	1.673	1.143–2.448	0.008^1^	2.038	1.436–2.891	0.0006^1^
recurrence	1.372	0.952–1.976	0.090			
Serum AFP	0.454	0.298–0.692	0.0002^1^	2.455	1.629–3.703	0.0001^1^

Abbreviations: HR, Hazard radio; CI, Confidence interval.

1 Statistically significant (*P*<0.05).

### The Expressions of miR-141 and Tiam1 in HCC Tissues and Cell Lines

**Figure 3:**
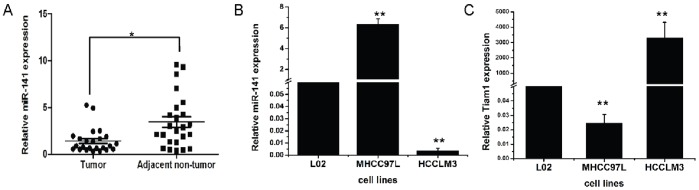
The expression levels of miR-141 and Tiam1 in HCC tissues and metastatic HCC cell lines. (A) MiR-141 mRNA expression in tumor (n = 30) and adjacent non-tumor (n = 30). (B) MiR-141 mRNA expression in LO2, MHCC97L and HCCLM3. (C) Tiam1 mRNA expression in LO2, MHCC97L and HCCLM3. The expression of miR-141 and Tiam1 was quantified by quantitative RT-PCR and normalized to U6 snRNA and β-actin, respectively. Data is presented as the mean ± SEM; *P* values were calculated using the Student’s t-test. *P<0.05,**P<0.01.

We examined the expression of miR-141 in 30 freshly frozen HCC tissues and adjacent normal tissues by using quantitative real-time polymerase chain reaction (qRT-PCR). Compared to the normal tissues, the expression of miR-141 was significantly down-regulated in HCC tissues (p = 0.01) (Figure 3A). In addition, we also analyzed the expression of miR-141 and Tiam1 in a panel of human HCC cell lines with different metastatic potentials but with similar genetic background. As presented in Figure 3B, the mature miR-141 was more abundant in lowly metastatic HCC cell line MHCC97L than in HCCLM3 that have high metastatic potential. Interestedly, the expression level of miR-141 in the two cell lines was negative associated with that of Tiam1 mRNA (Figure 3C).

### Alteration of miR-141 Expression Regulated the Proliferation, Migration and Invasion of HCC Cells in vitro

**Figure 4:**
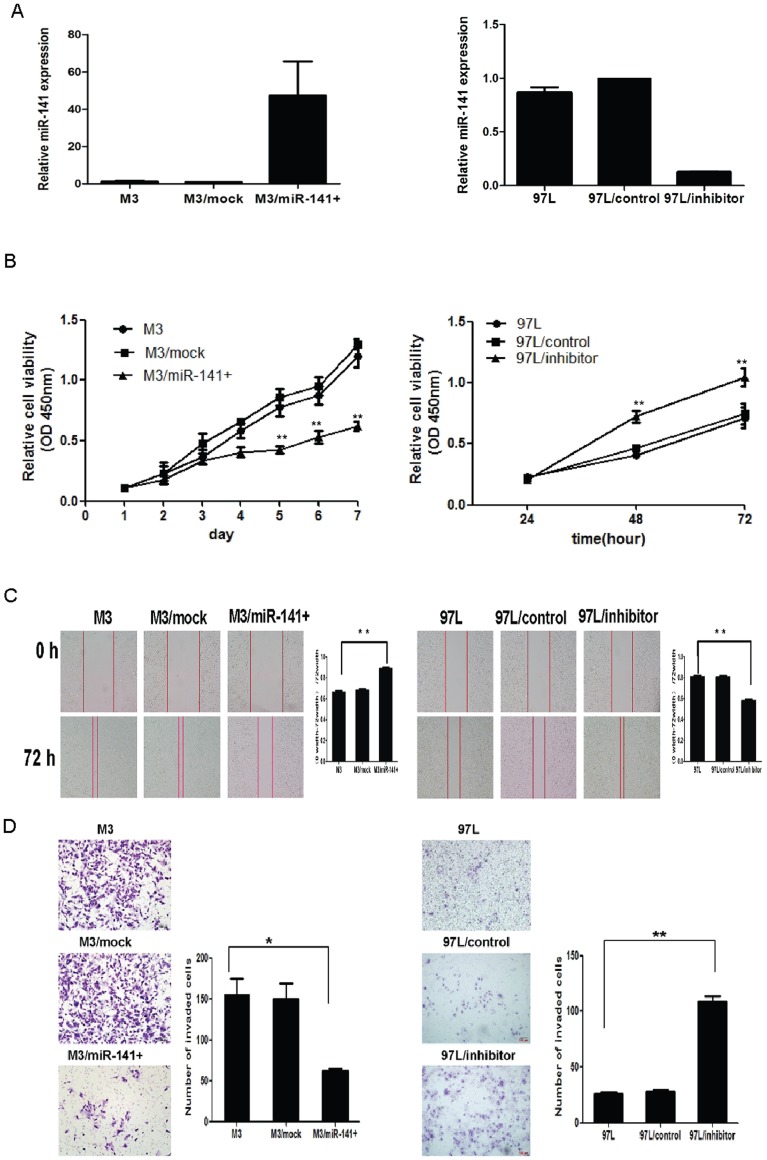
Effect of miR-141 on growth and invasion of HCC cell in vitro. (A) MiR-141 mRNA expression levels in M3 transfectant clones and knockdown cell relative to control. (B) Up-regulation of miR-141 in HCCLM3 cells reduced inhibited cell proliferation, while inhibition of miR-141 expression in MHCC97L cells increased cells growth cell compared with the controls. (C) Representative images of Wound-healing assay. The wound healing rate in HCCLM3 cells transfected with miR-141 overexpressed lentivirus was significantly decreased, while accelerated in MHCC97L cells transfected with miR-141 inhibitor. (D) Representative images (left) and quantification (right) of the Transwell invasion assay. The number of invaded cells in the HCCLM3 cells was significantly decreased, while the number of invaded cells in the MHCC97L cells was significantly increased compared with control. Data is presented as the mean ± SEM; *P* values were calculated using the Student’s t-test. *P<0.05, **P<0.01.

To further investigate the biological significance of miR-141 in HCC, we transfected miR-141 over-expressed lentivirus or miR-141 inhibitor into human HCC cell lines that have different endogenous expression levels of miR-141. Expression of miR-141 was verified by qRT-PCR (Figure 4A, left). CCK-8 assay manifested that up-regulation of miR-141 in HCCLM3, which have high metastatic potential and low endogenous miR-141 expression levels, resulted in significant suppression of cell proliferation (Figure 4B, left). Wound-healing assay showed that the mobility of M3/miR-141+ cells evidently decelerated in rate within 72 hr compared with the controls (Figure 4C, left). Transwell assay with matrigel showed that up-regulation of miR-141 resulted in a significant decrease in the invasive potential of HCCLM3 cells compared to control cells (p<0.05) (Figure 4D, left).

In contrast, the endogenous miR-141 level was knocked down by transfecting miR-141 inhibitor in MHCC97L (Figure 4A, right), which have low metastatic potential and high endogenous miR-141 levels. The results showed that down-regulation of miR-141 significantly increased cell mobility and proliferation compared with the negative control (Figure 4B, 4C right). Similarly, silencing of miR-141 obviously accelerated the invasion of MHCC97L cells (p<0.01) (Figure 4D, right). These results suggest that miR-141 regulates proliferation of HCC cells and significantly inhibits in vitro invasion of HCC cells.

### Tiam1-3′UTR is a Potential Functional Target of miR-141

**Figure 5:**
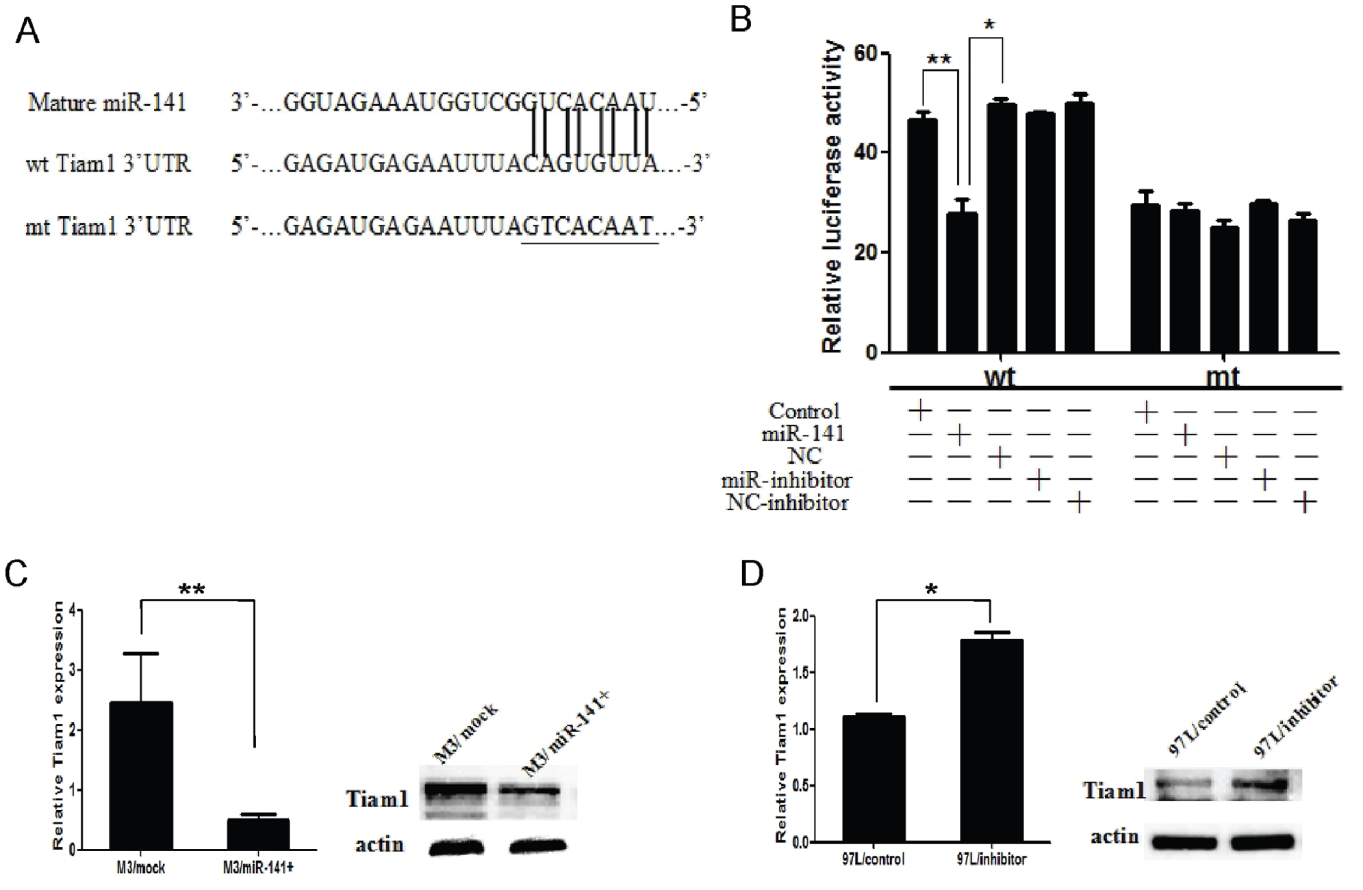
Tiam1 is a direct target of miR-141 in HCC. (A) miR-141 and its putative binding sequence in the 3′UTR of Tiam1. The mutant Tiam1 binding site was generated in the complementary site for the seed region of miR-141 (wt, wild type; mt, mutant type). (B) miR-141 significantly suppressed the luciferase activity that carried wt but not mt 3′UTR of Tiam1. (C) up-regulation of miR-141 significantly decreased both the mRNA and protein levels of Tiam1 in HCCLM3 cells compared with control. (D) down-regulation of miR-141 noticeably reduced both the mRNA and protein levels of Tiam1 in MHCC97L cells compared with control. Data is presented as the mean ± SEM; *P* values were calculated using the Student’s t-test. *P<0.05, **P<0.01.

To explore whether Tiam1 is the candidate target gene of miR-141, we used publicly available databases, including TargetScan (http://www.targetscan.org/), DIANA (http://microrna.gr/microT-ANN), and miRanda (http://www.microrna.org). According to TargetScan analysis, the 8mer complementary sequence of miR-141 was found in the 3′UTR of Tiam1 mRNA, leading us to further experimental validation. We cloned the wild-type or mutant sequences of the Tiam1 3′UTR into luciferase reporter vectors (Figure 5A). Our luciferase report showed that miR-141 significantly suppressed the luciferase activity of Tiam1 containing a wild-type 3′UTR but did not suppress activity of Tiam1 with a mutant 3′UTR (p<0.01) (Figure 5B), confirming that miR-141 can bind to the Tiam1 3′UTR. Next, we used quantitative RT-PCR and Western blotting to quantify endogenous Tiam1 mRNA and protein expression. Results showed that over-expression of miR-141 significantly reduced the mRNA level of Tiam1 in HCCLM3 cells and reduced Tiam1 protein levels in cell culture supernatant (p<0.01) (Figure 5C). In contrast, down-regulation of miR-141 significantly increased the mRNA and protein levels of Tiam1 in MHCC97L cells (p<0.01) (Figure 5D). Moreover, we analyzed the correlation between miR-141 and Tiam1 expression in the 212 frozen HCC samples based on LNA ISH assay. Spearman’s correlation analysis showed that the expression of miR-141 was inversely correlated with Tiam1 expression in the clinical HCC samples (Table 3, r = −0.262, p<0.01). Taken together, these results strongly suggest that miR-141 can regulate the expression of Tiam1 in HCC by directly targeting the Tiam1 3′UTR.

**Table 3:** Crosstab showing the inverse correlation between miR-141 and Tiam1.

		Tiam1		Total
		Lowexpression	Highexpression	
miR-141	Low expression	43	97	122
	High expression	47	35	90
Total		90	122	212

Spearman correlation, r = −0.262, P<0.01.

### Alterations of Tiam1 Influence the Effects of miR-141 on HCC Cells

**Figure 6:**
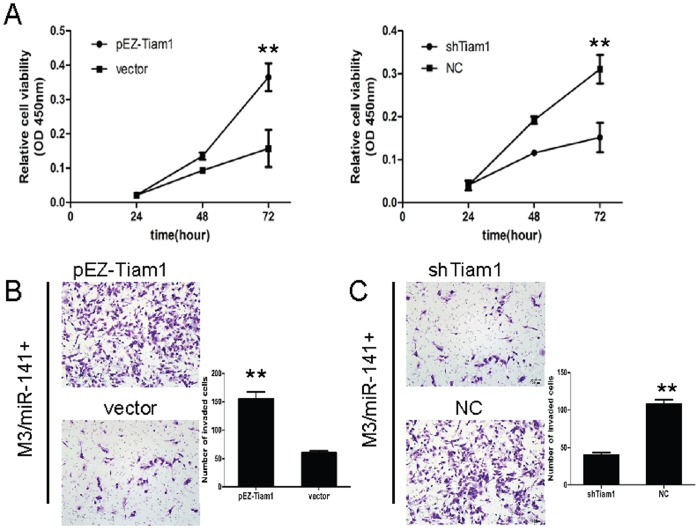
Alterations of Tiam1 influence the effects of miR-141 on HCC cells. (A) Representative images of the proliferation assay. Up-regulation of Tiam1 in M3/miR-141+ cells induced cell proliferation (left), while knockdown of Tiam1 suppressed cell proliferation (right). (B), (C) Representative images (left) and quantification (right) of the Transwell invasion assay. The number of invaded cells in the M3/miR-141+ cells treated with Tiam1 was significantly increased (B), while suppression of Tiam1 induced effects that were similar to those stimulated by miR-141(C). Data is presented as the mean ± SEM; *P* values were calculated using the Student’s t-test. **P<0.01.

To further confirm Tiam1 is a functional target of miR-141, we infected the HCCLM3 cell line stably overexpressing miR-141 with pEZ-M02 vector or pEZ-M02-Tiam1 plasmid, which encoded the full-length coding sequence of Tiam1 without its 3′UTR. The proliferation assay and the Matrigel Transwell assay showed that Tiam1 treatment significantly increased the HCC cell proliferation and invasion (Figure. 6A, P<0.01). In contract, Tiam1 short hairpin RNA (shTiam1) significantly inhibited cell proliferation and invasion (Figure. 6B and C, P<0.01). These results provide further evidence supporting Tiam1 as a functional target of miR-141 in HCC.

## Discussion

Until now, several researches have demonstrated that dozens of miRNAs are involved in HCC development and aberrant expression of some specific miRNAs can be used as a prognostic indicator for HCC patients [26]. MiR-141, which belongs to the miR-200 family, was found to be a useful biomarker for the diagnosis of liver malignancies [27]. However, little is known about the in vivo localization of miR-141 in human HCC tissue samples. In this study, we detected the miR-141 expression in the 212 HCC samples that were used in our previous study and analyzed the possible predictive value of miR-141 in patients with HCC based on ISH analyses. This method has recently demonstrated its reliability in predicting prognosis in colon cancer patients [28]. The Kaplan-Meier survival analysis revealed that low expression of miR-141 significantly correlated with a poor prognosis of HCC patients after surgical resection. Furthermore, multivariate Cox regression analysis demonstrated that low miR-141 expression was an independent prognostic factor for poor survival in HCC. Our previous study has shown that over-expression of Tiam1 was associated with decreased disease-free survival of patients with HCC [20]. Taken together, these findings may imply that miR-141 in combination with Tiam1 could improve the accuracy of predicting which HCC individuals may have a poor prognosis. In addition, our contingency table analysis (χ^2^ test) showed that miR-141 expression was associated with metastasis, which indicates that miR-141 could serve as a useful tool to identify HCC patients at high risk of metastasis. Although several researches have reported the association of miR-141 expression with different carcinoma, the results lack consistency. MiR-141 was found to be up-regulated in ovarian carcinoma [29], colorectal carcinoma [30], nasopharyngeal carcinoma [31], prostate cancer [32] and down-regulated in renal cell carcinoma [33], gastric cancer [34] and breast cancer [35]. These opposing findings substantiate the hypothesis that miR-141 may play different roles as an oncogene or a tumor suppressor gene in different cancer types. To our best knowledge, however, our observations have not been previously reported. Therefore, our data provided a more comprehensive understanding of the role of miR-141 in HCC.

Our previous studies have demonstrated that Tiam1 expression correlated with metastasis [21]. However, little was known about how this GNEF is regulated in HCC. To increase the specificity, in this study we first used three computational prediction tools: miRanda, TargetScan and DIANA, which predicated that Tiam1 is a potential function target of miR-141. We then found that miR-141 expression in HCC tissues correlated inversely with Tiam1 expression. Moreover, the ability of miR-141 to target Tiam1 was favored by the observation that inverse correlation was observed between miR-141 and Tiam1 expression in two HCC cell lines of different metastatic potential (see “Material and Methods” section). To determine whether miR-141 control Tiam1 expression, we established HCCLM3 cell over-expression of miR-141, and then examined the ability of cell proliferation, migration and invasion. Studies showed that the introduction of miR-141 significantly inhibited proliferation, migration and invasion in M3/miR-141+ cells compared with controls. Furthermore, we found that over-expression of miR-141 could significantly down-regulate the protein and mRNA level of Tiam1. In contrast, knockdown of miR-141 in MHCC97L cells resulted in significant increases in cell proliferation, migration and invasion. Similarly, the protein and mRNA level of Tiam1 were up-regulated. Therefore, we further confirmed Tiam1 was a directly functional target of miR-141. The dual-luciferase reporter assays indicated that Tiam1 was one of the functional downstream targets of miR-141, which suggested that miR-141 suppressed Tiam1 expression by interacting with the 3′UTR of Tiam1 mRNA. Moreover, ectopic expression of Tiam1 significantly increased the proliferation and invasion of HCCLM3 stably overexpressing miR-141, and knockdown of Tiam1 induced effects that were similar to those stimulated by miR-141. These results demonstrate that Tiam1 is a functional target gene of miR-141 in HCC. MiR-141 was also reported to target SIP1 in colorectal cancer [36] and CDC25B in renal cell carcinoma [37]. In other words, the reported targets of miR-141 and our findings indicated that miR-141 might regulate multiple signaling pathways, and loss of miR-141 would lead to the tumor progression in HCC.

It was well-known that metastasis is associated with poor prognosis, and therefore targeting its mechanism may lead to more effective treatment for HCC patients. The ability of short RNA sequences to modulate gene expression makes them very attractive for drug development. Lentiviral vectors can infect not only dividing cells but also non-dividing ones, which provide efficient gene delivery in vitro. Until now, lentiviral vectors encoding miRNAs have been universally used to study gene functions and some are currently being considered for clinical gene therapy applications [38], [39]. The above finding highlights the possibility of miR-141 as a novel target for therapeutic intervention.

In conclusion, our study indicates that miR-141 inhibits liver cancer cells by negatively regulating the Tiam1 gene. Our findings also underscore the clinical potential of miR-141 in HCC treatment and support the development of effective therapeutic strategies that target miR-141 (or its targets such as Tiam1) by a genetic or pharmacological approach.
